# Icaritin reduces prostate cancer progression via inhibiting high-fat diet-induced serum adipokine in TRAMP mice model

**DOI:** 10.7150/jca.48413

**Published:** 2020-09-21

**Authors:** Xiaobo Wu, Xingbo Long, Chen Yang, Huan Chen, Christina Sharkey, Khalid Rashid, Mengbo Hu, Yufei Liu, Qi Huang, Qi Chen, Jimeng Hu, Haowen Jiang

**Affiliations:** 1 Department of Urology, Huashan Hospital, Fudan University, Shanghai, China.; 2 Department of General Surgery, Division of Urology, Beth Isreal Deaconess Medical Center, Harvard Medical School, Boston, Massachusetts, USA.; 3 Fudan Institute of Urology, Huashan Hospital, Fudan University, Shanghai, China.; 4 Department of Urology, Beijing Hospital, National Center of Gerontology, Beijing, China.; 5 Department of Medicine, Beth Israel Deaconess Medical Center, Harvard Medical School, Boston, Massachusetts, USA.; 6 Department of Medical Oncology, Zhongshan Hospital, Fudan University, Shanghai, China.; 7 PET center, Huashan Hospital, Fudan University, Shanghai, China.; 8 National Clinical Research Center for Aging and Medicine, Fudan University, Shanghai, China.

**Keywords:** Icaritin, prostate cancer, TRAMP, adipokine, high-fat diet

## Abstract

**Objective:** Obesity resulting from high-fat diets has a close relationship with the morbidity and mortality associated with Prostate cancer (PCa) in males. The anti-cancer role of Icaritin (ICT, a traditional Chinese herbal medicine) has been reported in several types of cancer including PCa. Adipokines are novel adipocyte-specific secretory protein, which plays a key role in the development of various diseases including obesity, diabetes, atherosclerosis, and cancer. However, the function of ICT and the molecular mechanisms underlying its role in PCa regression through modulation of adipokines have not been studied. Here, we assessed the anti-cancer properties of ICT under the influence of human epidermal growth factor receptor type 2 (HER2) pathway modulating adipokines in obese PCa models.

**Materials and Methods:** In this study, we used transgenic adenocarcinoma of mouse prostate (TRAMP), a well-established animal model for the study of PCa pathogenesis. All the animals were fed on a high-fat diet (HFD with 40% fat) and divided into two groups, one received ICT solution of 30 mg/kg body bwt (i.p) while the other group served as control without any ICT treatment. The mortality rate, tumor formation and fat ratio were assessed by histopathological and magnetic resonance analysis at different time points of 20^th^, 24^th^ and 28^th^ weeks. The protein expression of HER2 and serum levels of adipokines were measured using western blotting, IHC and multiplex immunoassays. The PCa grade in 12 TRAMP mice were longitudinally evaluated to visualize PCa development and progression upon post-surgery using PET/CT scanning.

**Results:** We observed that ICT treatment significantly reduces the total mortality rate of TRAMP mice (*p* = 0.045) and the percentage of prostate intraepithelial neoplasia (PIN) or PCa (*p* = 0.029). Interestingly, significantly decreased levels of leptin (*p* = 0.006 @20^th^ wk) and the elevated levels of adiponectin (*p* = 0.030 @20^th^ wk) were observed in different subgroups upon ICT treatment in a time-dependent manner. In addition, a decrease level of HER2 (*p* = 0.032 @28^th^ wk) and an elevated level of PEA3 (*p* = 0.014 @28^th^ wk) were also detected in ICT treated group. The PET/CT-based imaging showed that ICT vs non-ICT treated mice had different standard uptake value and metastasis.

**Discussion and Conclusion:** Our results showed potent anti-cancer properties of ICT through the modulation of adipokine secretion may alter the expression and activation of HER2 pathway as an alternative mechanism to prevent PCa progression. Altogether, our findings indicate that ICT could be a promising cancer preventive agent with the potential to target and eradicate tumor cells in obese PCa patients.

## Introduction

Prostate cancer (PCa) has been considered as one of the most frequently diagnosed malignancies among male patients in Western countries 1, 2. High-fat diet (HFD) is one of the major characteristics of western eating style, which may alter molecular and cellular activity of various biological processes associated with inflammation and leading progression of various types of cancers, including PCa 3, 4. Earlier studies have shown that HFD may increase the incidence and mortality of PCa through the adipose-mediated secretion and production of numerous growth factors, cytokines, chemokines as well as hormone-like molecules 5-7. Furthermore, it has also been observed that PCa progression is closely related to abnormal secretion of adipokines and cytokines in overweight patients 8, 9. In line with the previous findings, it has been demonstrated that leptin and adiponectin are the two primary components among all adipokines. The elevated levels of serum leptin are associated with tumor progression, while the adiponectin diminishes the proliferation of tumor cells 10.

TRAMP animals are regarded as the most widely applied mouse models for the investigation of molecular and cellular alterations associated with PCa, as they closely resemble the development and progression of human PCa 11. In our previous findings, we have demonstrated that TRAMP mice are the best-suited model to explain the molecular mechanisms underlying the role of HFD-induced PCa progression. In addition, we have found that HFD increases the mortality rate of TRAMP mice 12, and potentiate PCa development and progression through elevation in adipokine and proinflammatory cytokine levels 13, 14.

Icaritin is a hydrolytic product of Icariin, a traditional Chinese herbal medicine extracted from the *Epimedium genus.* Interestingly, in our previous findings, we have shown anti-inflammatory properties of ICT as it significantly inhibits the secretion and production of serum proinflammatory cytokines, including interleukins (IL-1α, IL-1β, IL-6) and tumor necrosis factor-alpha (TNF-α) in TRAMP mice 15, 16. However, the anti-cancer role of ICT in the regulation HFD-induced adipokines mediated PCa progression has not been reported yet.

In the present study, we analyzed the effects of ICT treatment on the regulation of different adipokines. Through a series of repeated experiments, we found that ICT inhibits HFD-induced PCa progression via regulation of serum adipokines sercretion. Our results indicate a strong rationale for the potential clinical application of ICT as a safe and novel oncotherapeutic agent against PCa progression in obese patients.

## Materials and methods

### Animals and diets

TRAMP mice were purchased from Jackson Laboratory (Bar Harbor, Maine, USA). Generation of transgenic mice, extraction of mouse-tiptoe DNA, and PCR based screening assay were accomplished 17. Mice were raised separately in a cage on cork dust bedding with a 12 hr light/dark cycle at room temperature of 22‑26 °C and humidity of 50-60%. A total of 42 TRAMP mice were randomly separated into two groups and the mice were fed on HFD with/without ICT treatment ad libitum at 5 weeks of age, respectively. As illustrated in Table 1, normal diet consisted of 20% from proteins, 16% calories from fats and 64% from carbohydrates, while HFD comprised 20% from proteins, 40% calories obtained from fats and 40% from carbohydrates. Mice diets were provided by Puluteng Mouse Diet Co., Ltd (Shanghai, China). All animal studies were approved by the Institutional Animal Care and Use Committee from Department of Laboratory Animal Science, Fudan University (Shanghai, China).

### ICT administration

ICT was purchased from Shanghai Win Herb Medical Science Corporation (China) and was dissolved in DMSO solution (Sigma, St. Louis, MO, USA). The concentration of DMSO in the working solution of ICT (purity of up to 99.5%) was restricted below 0.1% of the total medium volume. In experimental group (HFD with ICT), the ICT solution dosage was set at 30 mg/kg based on previous experiments 15. ICT was administered to each TRAMP mouse intraperitoneally one time/day and 5-times/week randomly among week, starting from 8 weeks of the age until the completion of the respective experiments (20^th^, 24^th^ and 28^th^ week).

### Systemic evaluation, serum and tissue preparation

TRAMP mice from both groups were divided into three subgroups, which were euthanized and sampled on the 20^th^, 24^th^ and 28^th^ week by asphyxiation of CO_2_, respectively. TRAMP mice were required to fast overnight before being anesthetized with CO_2_. The bodyweight of each TRAMP mouse was evaluated before sampling. Blood (nearly 1 ml of each mouse) was collected from the portal vein by using 1 ml sterilized syringe. Subsequently, blood was centrifuged at 13,000 rpm for 10 min at 4 °C, and serum was collected in Eppendorf tubes. Both serum and prostate tissues were kept frozen at -80 °C for further analysis.

### Body fat ratio evaluation

The evaluation of body fat ratio of each TRAMP mice was performed before sacrifice by using minispec magnetic resonance analyzer (Bruker Corporation, Billerica, MA, USA) provided by Institute of Development Biology and Molecular Medicine of Fudan University (Shanghai, China).

### Histology studies

Prostate and other tissues were fixed in 10% buffered formalin, processed in alcohol-xylene and finally embedded in paraffin. A series of 2µm sections were prepared and stained with hematoxylin and eosin (H and E). For evaluation of tumor differentiation, these sections were additionally analyzed and cross-checked by two pathologists from Huashan Hospital, Fudan University (Shanghai, China). Histological sections were analyzed by light microscopy at a magnification of ×100 and ×400 to distinguish various PCa grades, which was classified as atrophic glands only (no identifiable tumor), prostate intraepithelial neoplasia (PIN) and PCa. In addition, we also performed the immunohistochemistry of HER2 in tumor tissues from each mouse after HE staining. Briefly, 2-mm tissue sections were incubated in EnVision Flex Target Retrieval Solution, pH low (Dako/Agilent, Santa Clara CA) followed by incubation of primary antibodies against HER2 (Cell Signaling Technology, Danvers, MA, USA 1:500) at room temperature for 20 minutes. Polymeric secondary antibodies coupled to horseradish peroxidase (EnVision Flex+, Dako, Glostrup, Denmark) and DAB (Dako) were applied to visualize the sites of immunoprecipitations. Tissue samples were analyzed by light microscopy after counterstaining with Meyer's hematoxylin. Staining intensity was evaluated using a 3-tiered semiquantitative scoring system: 0 = negative; 1 = weakly positive; 2 = strongly positive.

### Serum analysis

Leptin and adiponectin content (pg/mL) in mouse sera was evaluated using commercial ProcartaPlex Multiplex Immunoassays (Thermo Fisher Scientific, Inc., Waltham, MA, USA), which combine Luminex technology with Multi-Analyte Profiling beads and enable the simultaneous investigation and quantification of multiple cytokines in one sample. A total of 30 µl serum from each mouse was measured according to the manufacturer's protocol.

### Western blot

Antibody for PEA3 (ab70425) was purchased from Abcam Ltd (Shanghai, China). Anti-HER2 (#2165) was obtained from Cell Signaling Technology (Beverly, Massachusetts, USA). Protein from tumor tissues was extracted and concentrated using RIPA lysis buffer containing protease inhibitors (Sigma-Aldrich, St Louis, MO, USA) and the BCA Protein Assay kit (Vigorous Biotechnology Beijing, Beijing, China), respectively. Protein (20 μg) was resolved by SDS-PAGE, then transferred onto nitrocellulose membranes (Millipore, Madison, WI, USA). Membranes were blocked with 5% nonfat dry milk for 2 hours before being incubated with primary antibodies at 4 °C overnight. GAPDH was used as internal control. Membranes were then incubated with HRP-coupled secondary antibodies for 1 hour at room temperature. Image J (Version 1.8, National Institutes of Health, Bethesda, Maryland, USA) was used to quantify the grey value of each band. Data were normalized to GAPDH loading controls and statistically compared afterward.

### PET/CT analysis

A group of 12 TRAMP mice was evaluated *in vivo* with [^18^F]FDG-PET/CT approximately every 4 weeks starting from 20 weeks of the age. [^18^F]FDG studies were performed as followed: The 10 ±1.0MBq of the tracer were injected in a tail vein of different animals. Ten minutes before radioligand administration, animals were anesthetized, as described earlier. Just before the image acquisition, mice were positioned supine on the tomographic bed and their abdomen centered on the tomographic field of view. PET scanning was started at 60min after tracer injection and lasted for 30 min by Inveon PET/CT machine (SiemensInveon, Bonn, Germany). The standard uptake value (SUV) of region of interest (ROI) was analyzed by Inveon Research Workplace (V2, SiemensInveon, Bonn, Germany).

### Statistical analysis

Data were expressed as mean ± standard deviation (SD). Two-sample *t*-test was applied for comparison of leptin and adiponectin levels among different groups. Fisher's exact test or two-way ANOVA were used for comparison of categorical variables. Each TRAMP mouse was randomized into a different group in a blinded manner. All statistical analyses were carried out by using SPSS 23.0 for windows (Chicago, IL, USA) and GraphPad Prism 7 (version 7.0a; GraphPad Software, La Jolla, CA, USA ). *P* values less than 0.05 were considered to be statistically significant.

## Results

### Effect of ICT treatment on mortality and tumor formation rate in TRAMP mice

We observed only one death of TRAMP mouse in HFD group treated with ICT in comparison to seven deaths of TRAMP mice in HFD group without ICT treatment. The mortality rate in untreated HFD groups was significantly higher than that of HFD groups treated with ICT (Table 2, *P* = 0.045).

**Figure 1:**
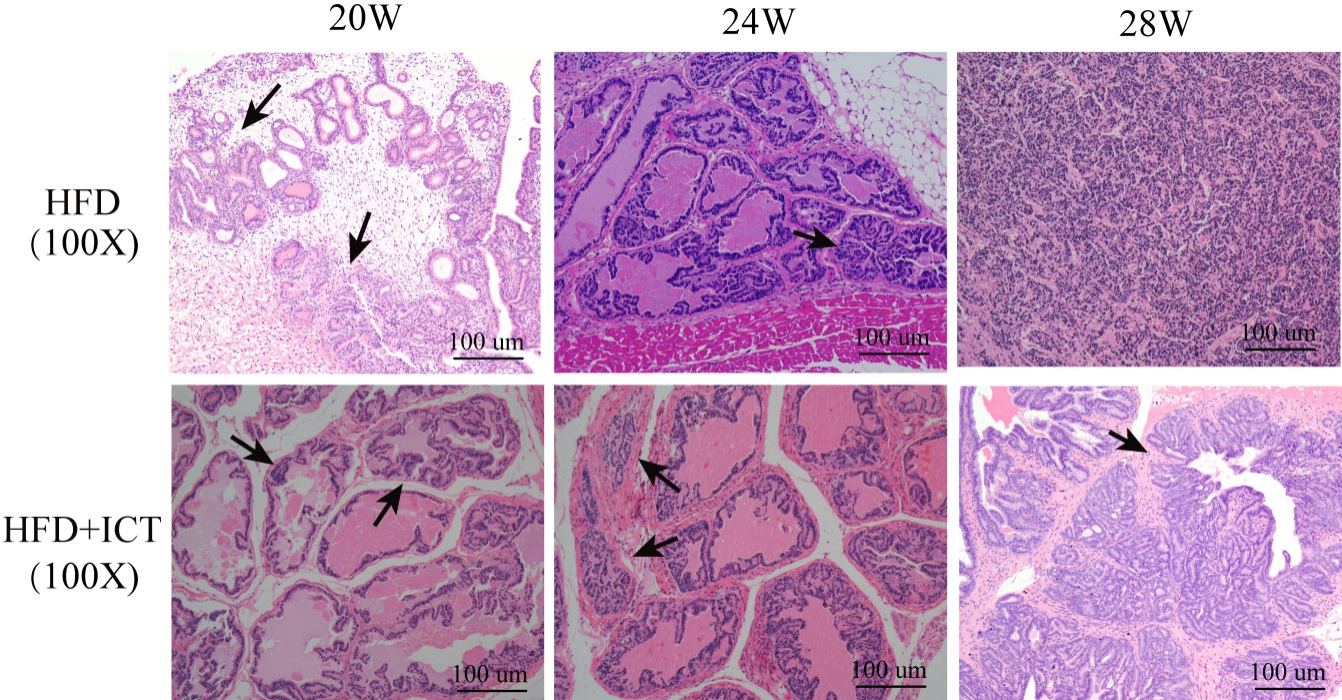
H & E staining of prostate acini from HFD and HFD with ICT at 20, 24 and 28 weeks of age, respectively. Arrow: Morphologically well-differentiated neoplastic glands. HFD+ICT group (@20th wk) showed normal prostatic gland morphology; HFD group (@20th wk) showed prostate intraepithelial neoplasia; HFD+ICT group (@24th wk) showed atrophic gland; HFD+ICT group (@28th wk) showed well-differentiated PCa; HFD group (@24th wk) showed moderated and HFD group (@28th wk) showed poorly diiferentiated PCa.

Supporting this finding, pathological examinations revealed a significantly higher percentage of PIN or PCa in the untreated HFD group than in HFD group treated with ICT in 20^th^ week subgroup (Table 3, *P* = 0.029). However, no significant difference was observed in either 24^th^ or 28^th^ week subgroup. Fig. 1 illustrated the proliferation of PCa cells in untreated HFD group and HFD group treated with ICT at 20, 24 and 28 weeks of age by means of H&E staining, respectively.

### Effect of ICT treatment on body weight and fat ratio in TRAMP mice

**Figure 2:**
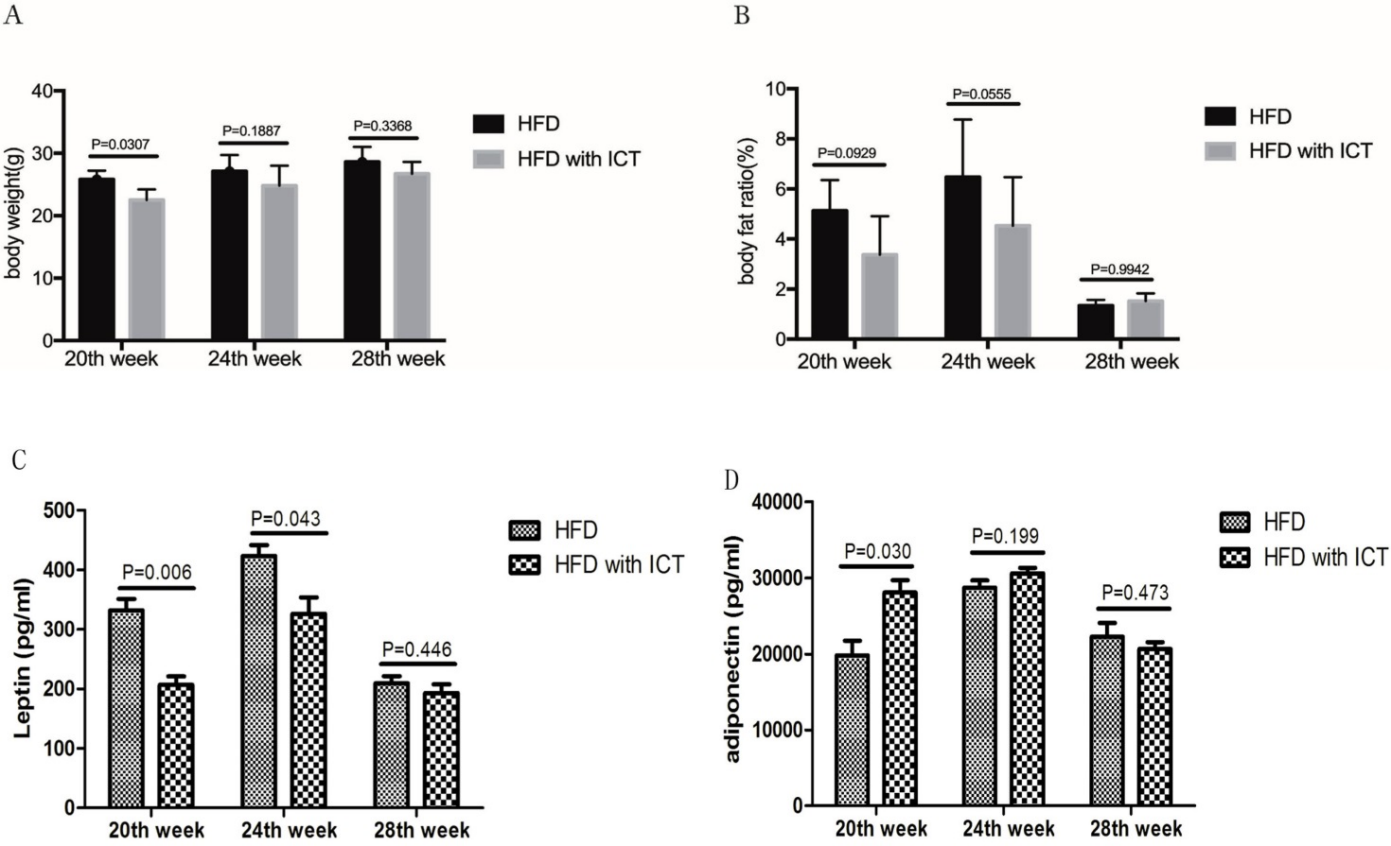
(A) Body weight (g) and (B) fat ratio (%) of different weeks old TRAMP mice in HFD group and HFD with ICT group. Two-way ANOVA analysis was used for comparison between HFD and HFD with ICT group. Adipokines levels in TRAMP mice. Two-sample t-test was used for comparison of cytokine levels between HFD and HFD with ICT group; (C) Serum leptin level in TRAMP mice. D. Serum adiponectin level in TRAMP mice.

We observed higher body weights (*P* = 0.0307) in untreated HFD group compared with HFD group treated with ICT in 20, 24 and 28^th^ week, respectively (Fig. 2A). However, the body fat ratio markedly decreased in 28^th^ week subgroups of both the untreated HFD group and HFD group treated with ICT in TRAMP mice (Fig. 2B).

According to the above pathological results, we speculate that the rapid decrease of the body fat ratio may be due to the process of cachexia and the abnormally elevated lipolysis and fat consumption occur due to the progression of PCa.

### Effect of ICT treatment on leptin and adiponectin levels in HFD mouse sera

Intriguingly, we have detected serum level changes of two major adipokines, leptin and adiponectin, which play a key role in the HFD-mediated PCa progression. A significant decrease of leptin was observed in TRAMP mice of HFD group treated with ICT at 20^th^ and 24^th^ weeks of age with a *P* value of 0.006 and 0.043 respectively. Moreover, the leptin level of HFD group treated with ICT at 28 weeks of age was lower than that of 20^th^ and 24^th^ week subgroups (Fig. 2C).

Adiponectin levels in TRAMP mice are shown in Fig. 2D. In contrast to leptin, a significant increase of adiponectin was observed in HFD group treated with ICT at 20 weeks of age (*P* = 0.030). Though TRAMP mice from HFD group treated with ICT at 24 weeks of age exhibit moderately higher adiponectin level than the untreated HFD group, it was not a significant differences (*P* = 0.199). Furthermore, we observed only marginal differences in the adiponectin levels in TRAMP mice of both the groups at 28 weeks of age (*P* = 0.473).

### Effect of ICT treatment on HER2 and PEA3 expression levels in prostatic tissue

**Figure 3:**
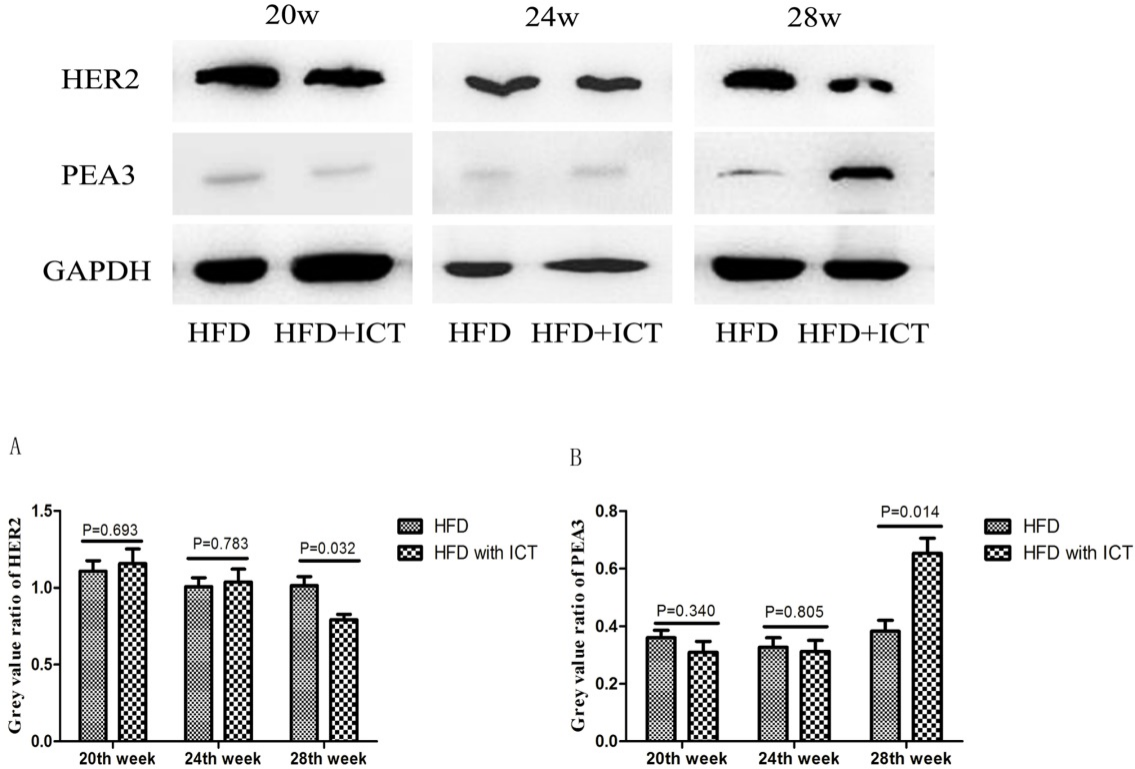
Western blot analysis of prostate HER2 and PEA3 in TRAMP mice at different weeks of age, n=7 mice per condition. Grey value ratio of prostate HER2 (A) and PEA3 (B) in TRAMP mice at different weeks of age, n=7 mice per condition. Two-sample t-test was used for comparison between HFD and HFD with ICT group.

**Figure 4:**
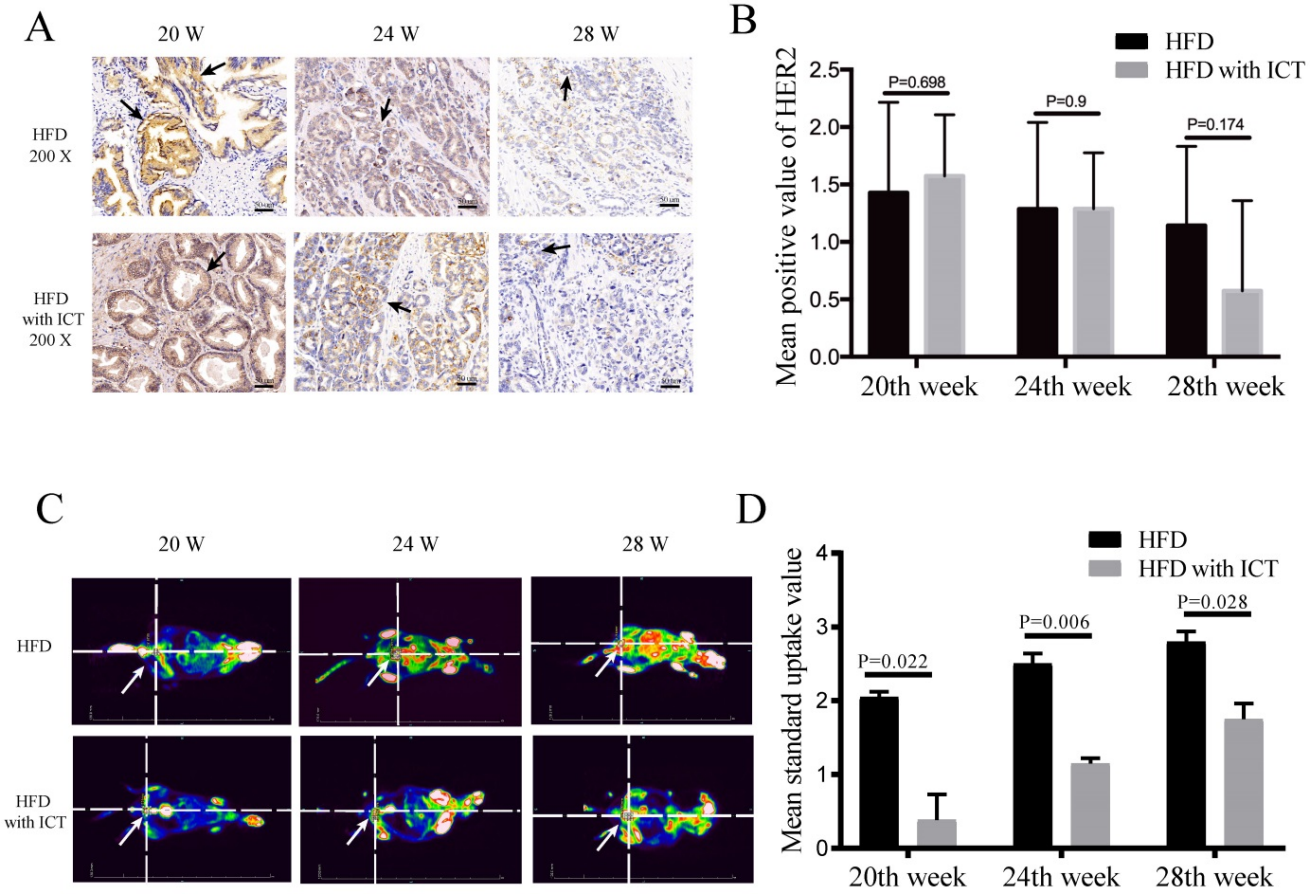
(A-B) Immunohistochemistry of HER2 at different weeks of age, n=7 mice per condition. Two-sample t-test was used for comparison between HFD and HFD with ICT group. Arrow: Positive staining region. (C-D) PET-CT analysis at different weeks of age, n=4 mice per condition and their mean SUV per condition. Two-way ANOVA analysis was used for comparison between HFD and HFD with ICT group. PET/CT showed that HFD groups had higher mean standard uptake value of Region of Interest (ROI) around/in prostatic area and more distant metastatic sites of prostate cancer than HFD with ICT group (red lighted area, Arrow: Indicate prostatic area).

The protein levels of HER2 and PEA3 in TRAMP prostatic tissues were detected by western blot. As shown in Fig. 3, we observed a significant decrease of HER2 level and a significant increase of PEA3 level in HFD group treated with ICT at 28 weeks of age. In addition, we have performed the immunohistochemistry of HER2 in TRAMP prostatic tissues from different groups (Fig. 4A). Staining intensity of HER2 was evaluated by pathologists using a 3-tiered semiquantitative scoring system: 0 = negative; 1 = weakly positive; 2 = strongly positive. Semi-quanitification of HER2 immunohistochemistry demonstrated a decrease of HER2 level in HFD group treated with ICT at 28 weeks of age, though there is no significant difference (*P* = 0.174) (Fig. 4B).

### PET-based imaging

The standard uptake value (SUV) and tumor metastasis of different groups of TRAMP mice in PET-based imaging suggested that they have different grades and progression of PCa. The ICT treated groups showed less metastatic sites of PCa (Fig. 4C). Moreover, a significant difference of SUV was observed in Region of Interest (ROI) between two groups at 20^th^, 24^th^ and 28^th^ week of age with a *P* value of 0.022, 0.006 and 0.028, respectively (Fig. 4D).

## Discussion

Recent studies revealed the anti-inflammatory and anti-survival activities of ICT in various cancer types including PCa 15, 16, 18-20. It is established that HFD-induced obesity is a well-known risk factor for the development of various diseases, including cancer, through regulatory mechanisms 2, 21. Our findings indicated that ICT can diminish both the tumor formation and mortality of HFD-fed TRAMP mice through reduction of leptin levels and elevation of adiponectin levels. After ICT treatment, we observed a significant decrease in both the body weight and fat ratio of 20^th^ week subgroup compared to the HFD-fed subgroup without ICT treatment. Our findings suggest that ICT may reduce prostate cancer progression via optimal regulation of adipokines in TRAMP mice.

Leptin is one of the potential pro-inflammatory adipokines that is mainly secreted in adipose tissues 22. In a normal healthy situation, leptin plays a key in the regulation of energy homeostasis. However, it may be involved in various physiological and pathological conditions such as proliferation, fibrosis, angiogenesis and metastasis via activation of various growth factors and signaling cascades in PCa 23, 24. Previous literature demonstrated that leptin receptor was found in normal, high-grade PIN lesions and malignant prostatic epithelium, respectively. Moreover, elevated levels of plasma leptin was detected in prostate cancer patients, which might have a positive correlation with PCa development 25. Leptin is found to be closely linked with several GF-receptors including HER2. It was demonstrated that HER2 can induce transcriptional activation of leptin in normal breast epithelial cells 26. Furthermore, leptin treatment increases HER2 protein levels in breast cancer cells 27. In this study, we found a significant decrease in serum leptin levels in both 20^th^ and 24^th^ week subgroups after ICT treatment. This trend of leptin level was consistent with the alterations in the level of body weight and fat ratio in TRAMP mice. Thus, we consider that the noticiable decrease of serum leptin in 28^th^ week may be due to the constant consumption of adipose tissues during the progression of PCa.

Adiponectin is another potent adipokine, produced by adipocytes, which is involved in various metabolic activities, including cancer. The low level of adiponectin was found associated with a significantly higher risk of PCa 28. Bub et al showed that adiponectin could inhibit the proliferation of PCa cells 29. Our results illustrated a significant increase in adiponectin levels in 20^th^ week subgroup after ICT treatment. Surprisingly, no statistical difference was observed in either 24^th^ or 28^th^ week subgroup. These results indicated that ICT could inhibit serum leptin levels and up-regulate adiponectin levels in the early stage of PCa development and thus suppressed its progression.

Human epidermal growth factor receptor type 2 (HER2) is a receptor tyrosine kinase, which has been detected in 17-22% of PCa tissues in a previous retrospective study 30. Moreover, polyomavirus enhancer activator 3 (PEA3), a member of the ETS transcription factor family, could specifically bind to the HER2 gene promoter and down-regulate its activity 31, 32. Evidence showed that HFD-induced obesity could be one of the reasons to develop tumor in HER2/Neu mouse model of breast cancer 33. It was also noted that the development of tumor is faster in HER2/Neu mice fed on HFD, possibly due to alteration in adipokines/cytokines. Several studies have suggested that obesity is correlated with larger tumors, advanced grade, HER2+subtype, and worse overall survival rate in breast cancer^34^, ^35^. HER2-overexpressed subtype of breast cancer exhibits significantly higher levels of the fat mass and obesity-associated (FTO) gene 36. The blockade of fatty acid synthase (FAS) negatively regulated the HER2 expression, indicating the link between FAS activity/fat metabolism (obesity) and HER2/PEA3 expression in the malignant transformation 37-39.

Interestingly, our previous study demonstrated that ICT exerts its potent anticancer efficacy by inducing PEA3 to inhibit the aberrantly activated HER2 signaling in LNCaP PCa cells 16. Consistent with our previous findings, we found that PEA3 protein level was relatively low in HER2-overexpressing TRAMP prostatic tissues. After ICT treatment, PEA3 protein level was found to be up-regulated, while level of HER2 declined at a significant level in 28^th^ week of subgroup. The changes were observed in western blot (WB), but not in immunohistochemistry (IHC).

Our findings suggest that the significant impact of ICT on the adipokines (leptin and adiponectin) occurs at 20^th^ week, while the significant alterations in the expression of HER2/PEA3 were observed at 28^th^ week. The change could be due to the depletion of adipose tissues and the reduction of adipokines level in response to elevated cachexic effect at 28^th^ week of tumor progression. Our data shows slight adipokine alterations effects by ICT on HER2 expression at 20^th^ week. However, these effects were not as prominent compared to the effects at 28^th^ week. The delay in response may be due to the difference in the biological properties of these two proteins:adipokines are secretory proteins while HER2 is a membrane-bound protein. In addition, defects/hindrance in the transportation/translocation of HER2 to either plasma membrane or nucleus can also affect the expression levels. HER2 can also modulate the transcriptional activity of many genes associated with different signaling cascades including adipokines in a bidirectional manner. Furthermore, circulating HER2 may also have some effects on the expression levels. Taken together, it's difficult to determine a proportional induction and expression of HER2 according to the levels of secretory adipokines in a time-dependent manner, but it could be a cumulative effect.

Although our findings showed a promising anticancer potential of ICT, there are limitations that exist in this study. First, the optimal dose and potential side effects of ICT were unknown. In future studies, we are planning to evaluate the impact of ICT on the prevention of metastatic growth rates in different organs of TRAMP mice, including prostate, liver, pancreas, and lungs. We will also investigate whether ICT could prolong TRAMP mice life span and investrigate the molecular mechanisms and signaling cascades that are involved in adipokine-mediated PCa progression. Lastly, we will determine the correlation between leptin, adiponectin, and PEA3/HER2 pathways to better understand the role of cascade interactions in PCa development. We will need to further examine the molecular properties of ICT to provide insights and novel therapeutic options for PCa treatment.

## Conclusion

In conclusion, our results suggest that ICT, a traditional Chinese herbal medicine from *Epimedium Genus*, inhibits the progression of PCa in TRAMP mice via blockage of high-fat diet-induced adipokines. It is necessary to point out that the efficacy of ICT is not yet confirmed in obese patients. However, based on findings of this study, ICT could be a promising agent in the suppression and prevention of PCa by regulating the expression and functions of major components of HFD-induced adipokines in obese patients, leptin and adiponectin.

## Figures and Tables

**Table 1:** Energy and nutrient composition of diets (gm %)

Diet	Normal diet	HFD
Protein	20	22
Fat	7	20
Carbohydrate	64	45
**Energy (Kcal/100 g)**		
Protein (%)	20	20
Fat (%)	16	40
Carbohydrate (%)	64	40

**Table 2:** Mortality rate of TRAMP mice

Characteristic	HFD Group (n=21)	HFD with ICT group (n=21)	*P* value
**Mortality**			0.045*
20^th^ week	1	0	
24^th^ week	2	0	
28^th^ week	4	1	
**n**	7	1	
Percentage (%)	33.33	4.76	

TRAMP transgenic adenocarcinoma mouse prostate, HFD high fat diet; * *P* < 0.05.

**Table 3:** Pathological results of TRAMP mice

Characteristic	atrophic	PIN	PCa	*P* value
HFD 20^th^ week	1	5	1	0.029*
HFD with ICT 20^th^ week	6	1	0	
HFD 24^th^ week	0	5	2	0.462
HFD with ICT 24^th^ week	2	3	2	
HFD 28^th^ week	0	3	4	N/A
HFD with ICT 28^th^ week	0	5	2	

TRAMP transgenic adenocarcinoma mouse prostate, HFD high fat diet, PIN prostate intraepithelial neoplasia, PCa prostate cancer; * *P* < 0.05.

## Abbreviations

PCa: prostate cancer
ICT: icaritin
HER2: human epidermal growth factor receptor type 2
TRAMP: transgenic adenocarcinoma of mouse prostate
HFD: high fat diet
PIN: prostate intraepithelial neoplasia
SUV: standard uptake value
ROI: region of interest
PEA3: polyomavirus enhancer activator 3
